# Spatial Organization of Five-Fold Morphology as a Source of Geometrical Constraint in Biology

**DOI:** 10.3390/e20090705

**Published:** 2018-09-14

**Authors:** Juan López-Sauceda, Jorge López-Ortega, Gerardo Abel Laguna Sánchez, Jacobo Sandoval Gutiérrez, Ana Paola Rojas Meza, José Luis Aragón

**Affiliations:** 1 Consejo Nacional de Ciencia y Tecnología (CONACYT, Mexico), 03940 Ciudad de Mexico, Mexico; 2 Área de Investigación de Sistemas de Información y Ciencias Computacionales, Universidad Autónoma Metropolitana, Unidad Lerma, 52005 Lerma de Villada, Mexico; 3 Centro de Física Aplicada y Tecnología Avanzada, Universidad Nacional Autónoma de México, 76230 Queretaro, Mexico

**Keywords:** pentagon, fivefold morphology, body plan, spatial organization, morphospace

## Abstract

A basic pattern in the body plan architecture of many animals, plants and some molecular and cellular systems is five-part units. This pattern has been understood as a result of genetic blueprints in development and as a widely conserved evolutionary character. Despite some efforts, a definitive explanation of the abundance of pentagonal symmetry at so many levels of complexity is still missing. Based on both, a computational platform and a statistical spatial organization argument, we show that five-fold morphology is substantially different from other abundant symmetries like three-fold, four-fold and six-fold symmetries in terms of spatial interacting elements. We develop a measuring system to determine levels of spatial organization in 2D polygons (homogeneous or heterogeneous partition of defined areas) based on principles of regularity in a morphospace. We found that spatial organization of five-fold symmetry is statistically higher than all other symmetries studied here (3 to 10-fold symmetries) in terms of spatial homogeneity. The significance of our findings is based on the statistical constancy of geometrical constraints derived from spatial organization of shapes, beyond the material or complexity level of the many different systems where pentagonal symmetry occurs.

## 1. Introduction

Pentagonal symmetry is a remarkable property of some biological systems [1]. There are many notable examples of pentagonal symmetry in the members of some biological groups like Echinodermata, radiolarians and plants. In many cases, the five-fold symmetry is clearly displayed but, in some others, the radial symmetry is partially overprinted by bilateral symmetry. However, in this last case the body is still divided into five parts but a secondary bilateral symmetry is superimposed on the body plan. In a more precise definition, symmetry is a property of an object which is invariant to any of various transformations; including reflection, rotation or scaling [2]. Besides the relevance of symmetry, our main goal here is a theoretical contribution to understand the emergence of shapes in terms of spatial configurational elements using polygons as systems. Our path is a statistical analysis of spatial distributions of areas associated with stars and partitions of discs, either symmetrical or not. For this reason, here we broaden the study of symmetry to the understanding of five-fold organization (FO) which in terms of phenotypes, development and evolution can be considered as fivefold symmetry. These five-part unit arrangements are frequent and in an evolutionary biology context, such patterns and their emergence have traditionally been approached by developmental genetics, systematics and ecology as a biological consequence of selective and ontogenetical processes [3,4,5,6,7,8,9,10,11]. Although common in nature, there are few general comments on the extended frequency of FO in phenotype shape, with some important exceptions [2,12]. In a pioneering work, Breder [12] shows that FO is a basic pattern in many flowers, dicotyledons, echinoderms, the vertebrate body section, the distal ends of tetrapod limbs, and of the oral armature of priapulids. Breder concludes “Five-partness, where it appears, is held to with great rigidity, even when extensive evolutionary change has taken place. This does not seem to be the case to such a marked extent where other symmetries are concerned, as the coelenterates witness”. The reasons for the success of FO, where it has evolved, are not yet understood although some hypotheses have been formulated in sea urchins and flowers, either based on their functional, ecological role, developmental constraints [8,9,10,13,14,15] or such as those derived from mechanical models, [16] or mathematical models associating pentagonal symmetry with robustness and pattern formation [17]. However, even these hypotheses seem to be supported by a large body of evidence, they do not explain the occurrence and robustness of FO in all other remaining organismic and non-organismic entities such as molecules, cellular and inorganic organizations. Neither do those approaches consider the emergence of shapes in terms of spatial interacting elements nor in terms of spatial variability. Breder [12] suggested that the origin of the stability of the FO lies in the geometrical properties of the pentagon. Consequently, our proposal requires a discerning of the statistical geometric properties for pentagonal arrangements in contrast with other geometries.

Convex polygons are plane entities and their geometry restricts the way inner regions (considered as sub-entities defining areas) are partitioned (e.g., the star associated with the vertexes of a pentagon can generate five identical areas). Those surface regions distribute areas inside polygons and, with a proper measurement of spatial organization in 2D shapes, a quantitative parameter of that distribution can be determined. In fact, an important constraint in any spatial region may be the spatial homogeneity which we can understand as regularity and a statistical spatial organization argument of this constraint associated with FO is developed here. In a previous work, we considered quantitative spatial homogeneity among areas inside a region (a bounded polygon) as synonymous of regularity and the presence of disparity among areas as spatial heterogeneity [18]. Therefore, our definition of spatial heterogeneity is based on the unequal distribution of areas inside polygons; we propose a parameter to define quantitatively the spatial organization of inner polygonal elements around three main concepts: eutacticity, regularity and spatial heterogeneity. It has been shown in a previous work [19] that eutacticity is a parameter closely linked with regularity and it is a suitable measurement of spatial homogeneity and heterogeneity [18]. Eutacticity is sharply linked with regularity by considering that a given polygon, polyhedron and, in general, polytope can be associated with a star of vectors (pointing from the center to the vertices) and it has been demonstrated that stars associated with regular polytopes are eutactic [20]. That regularity is derived from measurements of variation of partitioning areas using the vertexes of polygons as star points linked with areas. In terms of spatial interacting elements, in regular polygons of *n* sides there are equal areas arranging around one centroid resulting in a homogeneity of areas. In contrast, irregular polygons have variations of spatial states (areas) which will rearrange the distribution implying spatial heterogeneity. We weighed spatial homogeneity-heterogeneity using eutacticity in FO looking for geometrical constraints which we contemplated would give us some advice about the preferential emergence of shape organizations. The spontaneous organization of individual blocks into ordered structures is ubiquitous in nature and found at all length scales, thus the shape and the quantitative nature of the building blocks becomes increasingly important [21,22]. The analysis of abstract entities, such as the geometry of these building blocks, into constituent elements and their degrees of interaction among internal parts represents a source of important information in terms of evolutionary constraints and evolvability [23]. According to this, the modularity of spatial components might be a natural phenomenon able to explain the emergence of shapes. It is assumed that systems are composed of individual elements or *modules* and knowledge of modules and their integration is important to realize some properties of the particular containing systems. Behind the deep essence of modularity, the concept of module can be a useful tool to understand the development of organisms or generic systems, for instance due to organizational principles of self-maintaining systems [24], or it may be an “evolved property” [23]. In this work, we study the frequency of FO in nature by using the concept of module in simple polygonal organizations. Intuitively, here, a module is a summation of particular elements from many polygons regarding spatial variability and it will depict non-trivial differences between shapes, in terms of spatial organization, inside a universe of shapes or star morphospace. A theoretical morphospace has been proposed as a geometric space of both existent and non-existent biological forms [25], and an important step towards the proof that spatial homogeneity is related with the high occurrence of FO, is the development of a morphospace of stars with different symmetries. The final goal in our research will be to show that FO is restricted to a particular zone of spatial homogeneity using; (a) eutacticity inside a morphospace of stars with different symmetries and; (b) a second experimental evidence devoid of eutacticity using a simulation of disc partitions regarding spatial variability. Accordingly, we claim that spatial organization besides star morphospace should be examined as an important way to understand how existing shapes emerge in the morphological context of phenotypes as generic spatial organizations apt to change without losing equity.

## 2. Methods

### 2.1. Statistics of Spatial Organization for Shapes Γ

To establish a proper measure of spatial organization we start by defining a shape *Γ*. A shape *Γ* is a set of spatial planar confined regions called sub-localities inside a locality $L_{i}$. Hence, a shape might be a regular or irregular polygon. In addition, we will see that each shape *Γ* can be associated to a star which, eventually, will be turned into a number (a set of area) that can be subject to statistical analysis. Our statistical analysis will be derived from localities and their sub-localities coming from constructions of shapes *Γ*. The main idea to establish the generic name of shapes *Γ* is because it is useful to define either shapes or numbers associated with shapes.

**Figure 1:**
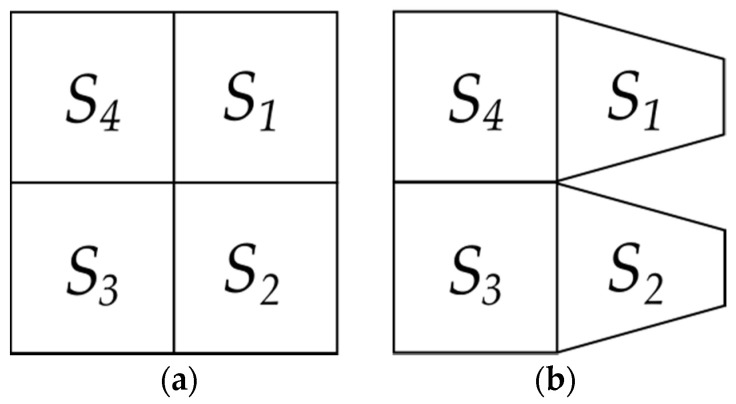
Schematic properties of two different shapes *Γ*. (**a**) A square is a locality associated to four subareas from four sub-localities $S_{1},S_{2},\ldots ,\;S_{4}$ which are all equal; (**b**) A shape *Γ* with a four-fold partition such that any of their sub-localities have unequal subareas is not regular; the set of areas defined by sub-localities $S_{1}$ and $S_{2}$ are smaller than those of $S_{3}$ and $S_{4}$.

Each locality $L_{i}$ is constituted by a subset of a given number $N_{i}$ of sub-localities, $S_{i1},S_{i2},\ldots ,S_{i_{Ni}}$ such that $L_{i}=\cup_{j=1}^{N_{i}}S_{ij}$, where $L_{i}$ is a convex regular or irregular polygon in $R^{2}$. Let $A_{ij}$ be the area of each sub-locality. If $A_{ij}=A_{ik}\;\forall \;k,j$, then we said that $L_{i}$ is regular (Figure 1). In contrast, if there exists some j≠k such that $A_{ij}\neq A_{ik}$ then we say that $L_{i}$ is not regular. Therefore, let $A_{i}=\overset{N_{i}}{\underset{j=1}{\sum }}A_{ij}$ be the sum of all the associated areas of every locality; this set determines $\Gamma =\left\{A_{i}\right\}$. Therefore, *Γ* is a generalization of locality or any set of sub-localities which will be understood as a number in statistical terms. Therefore, the area average of a locality $L_{i}$ is:

(1) $${\bar{A}}_{i}=\frac{1}{N_{i}}\overset{N_{i}}{\underset{j=1}{\sum }}A_{ij}$$

and

(2) $$\sigma_{i}=\sqrt{\frac{1}{N_{i}-1}\overset{N_{i}}{\underset{j=1}{\sum }}{\left(A_{ij}-\bar{A_{i}}\right)}^{2}}$$

is the standard deviation of each locality. Notice that if $\sigma_{i}=0\Rightarrow A_{ij}=A_{ik}\;\forall \;j,k$.

### 2.2. Mathematical Basis of Eutacticity

A star ψ is a set of n vectors $\left\{u_{1},u_{2},\ldots ,u_{n}\right\}$ with a common origin in an N-dimensional space $\left(R^{N}\right)$. The star is eutactic if it can be obtained by projecting an orthogonal set. Eutacticity is sharply linked with regularity by considering that a given polygon, polyhedron and, in general, polytope can be associated with a star of vectors (pointing from the center to the vertices) and it has been demonstrated that stars associated with regular polytopes are eutactic [26]. A good numerical criterion for obtaining the eutacticity of a star, suitable for dealing with experimental measurements, was proposed in Reference [26] and is as follows. Let B be the matrix whose N columns are the coordinates of the vectors forming a star ψ, with respect to a given fixed orthonormal basis of $R^{2}$. 

The star is eutactic if and only if:

(3) $$\varepsilon =\frac{Tr\left(S\right)}{\sqrt{Tr\left(SS\right)}\sqrt{2}}=1$$

where $S=BB^{T}$; Tr denotes the trace and the superindex T denotes the transpose. Notice that the parameter ε can indicate the degree of eutacticity of the star represented by B; if it is not strictly 1, which is the highest value of eutacticity, then the closer to 1, the more eutactic the star is. In case of planar stars, it can be proved that:

(4) $$\frac{1}{\sqrt{2}}\leq \varepsilon \leq 1$$

The strategy is to associate a given polygon or locality $L_{i}$, with a star *ψ*, using Equation (3) to measure its value of eutacticity. Next, a measure of spatial organization can be proposed and used to measure the regularity of a form *Γ*, using sub-locality areas. For this goal, we should prove that the closer ε is to 1, the more regular (the feature of spatial homogeneity) the star is (Section 2.3). Our hypothesis is that the higher the eutacticity, the more homogeneous (i.e., the area variability of the sub-locality decreases) the partition of the space is. Lower values of eutacticity imply unequal partition of the space, more area variability or spatial heterogeneity. According to Equations (1) and (2), the variability defining regularity must occur among localities. In order to support statistical differences between highly regular stars (highly eutactic stars), in contrast with non-regular stars (low eutectic stars), we need to define spatial variability between two experimental groups; highly eutactic and less eutactic stars using the values of polygons associated with them. 

### 2.3. The Eutacticity and the Standard Deviation of Dispersion Mean of a Module

**Figure 2:**
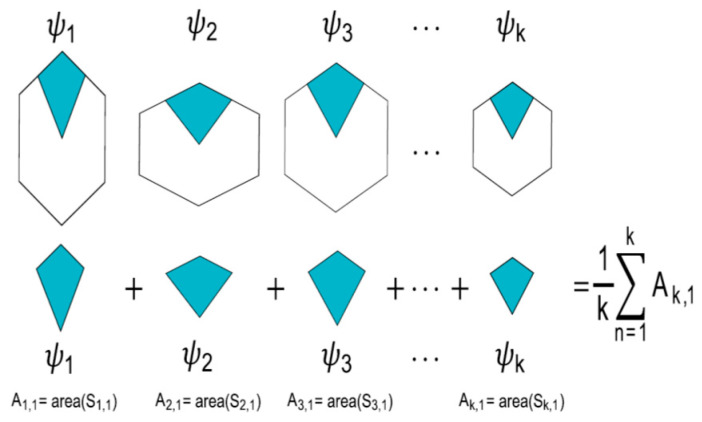
Construction of a module from k stars. A module is an average derived from an area summation of a particular sub-locality (e.g., sub-locality 1) from k stars ψ with a constant value ε. In this figure, the second sub index of *A* is referring to sub-locality 1. Stars $\psi_{1},\psi_{2},\ldots ,\psi_{k}$ are the building blocks to construct localities $L_{1},L_{2},\ldots ,L_{k}$. This process is applied to build modules of the two experimental groups of stars, $\psi_{a}$ and $\psi_{b}$.

The algorithms used in this section are found in Reference [27]. In this section, we will show that eutacticity is an important parameter to measure spatial organization. Here, we introduce the concept of module to support the statistical framework of Section 2.1, linking this with vector stars ψ described in Section 2.2. Spatial organization is the fundamental property to quantify regularity using polygons. A partition of the localities $L_{i}$ into sub-localities $S_{i1},S_{i2},\ldots ,\;S_{i_{Ni}}$ is proposed using Voronoi tessellations as proposed in Reference [27]. The goal in Reference [27] was to verify the spatial distribution of areas inside localities by comparing stars with high and low values of eutacticity. In this way, two experimental groups can be distinguished; $\psi_{a}$ representing eutactic stars (ε=1) and $\psi_{b}$ representing stars with a lower value of eutacticity (ε=0.8). With these two groups, we proceed as follows. There will be $\psi_{1},\psi_{2},\ldots ,\psi_{k}$ stars such that; (1) all of them have the same value of ε; (2) any of them has the same number of vectors ν; (3) they are geometrical random stars, even though any of them has the same eutacticity value (point 1). Finally; (4) Stars $\psi_{1},\psi_{2},\ldots ,\psi_{k}$ are the building blocks to construct localities $L_{1},L_{2},\ldots ,L_{k}$ with the number $N_{i}$ of sub-localities $S_{i1},S_{i2},\ldots ,S_{i_{Ni}}$ associated with the same number of vectors ν. In fact, according to property 2, we have $N_{i}=N_{j}=\nu ,\;\forall \;i,j$, which is an important condition to establish a formal definition of module. Intuitively, a module is a summation of particular sub-localities from many localities and it will be used to contrast two arbitrary values of ε numerically (Figure 2). 

According to Reference [27], let us assume that the areas $A_{i,j}$ associated to sub-localities of the two groups of stars ($\psi_{a}$,$\;\psi_{b}$) have two crucial components: (a) The eutacticity ε of the star ψ and (b) a set of random points $\omega_{m,n}$ defining the associated areas $A_{i,j}$. It is important to highlight that $L_{1},L_{2},\ldots ,L_{k}$ depend on $\psi_{1},\psi_{2},\ldots ,\psi_{k}$ (property 4 of stars ψ). According to this, $\psi_{1},\psi_{2},\ldots ,\psi_{k}$ are associated with $\omega_{m,n}$ which will define regions to establish sub-localities $S_{i1},S_{i2},\ldots ,S_{i_{Ni}}$. In that sense, let us call $\psi_{1,j}^{\omega_{1,n}},\psi_{2,j}^{\omega_{2,n}},\ldots ,\psi_{k,j}^{\omega_{k,n}}$ to the stars, where j represents the particular sub-locality and n is the set of random points n=1,…,α. So $\omega_{m,i}\neq \omega_{m,j}$ for every i≠j. In this case, m=1,…,k is a simple tag to associate star k with $\omega_{k}$ and subsequently with a set α of random points, and the associated areas are $A_{1,j}^{\omega_{1,n}},A_{2,j}^{\omega_{2,n}},\ldots ,A_{k,j}^{\omega_{k,n}}$. Therefore, the module for a particular sub-locality is defined using the average of its areas. Modules for particular sub-localities of two experimental groups of stars ($\psi_{a}$,$\;\psi_{b}$) are built in order to contrast its sub-locality area variations.

In Table 1, an example of the analysis of module 1, which is exclusive for sub-locality 1 in a locality of j sub-localities, is shown:

The summation Σ of module 1 derived from sub-locality 1 in a locality with j sub-localities, k stars and a set α of randomly generated points will be defined by:

(5) $$\frac{1}{\alpha }\left(\overset{\alpha }{\underset{n=1}{\sum }}A_{11}^{\omega_{1n}}+\overset{\alpha }{\underset{n=1}{\sum }}A_{21}^{\omega_{2n}}+\ldots +\overset{\alpha }{\underset{n=1}{\sum }}A_{k1}^{\omega_{kn}}\right)$$

Therefore, the average for module 1 is:

$${\bar{A}}_{\mu_{1}}=\frac{1}{\alpha k}\overset{k}{\underset{i=1}{\sum }}\overset{\alpha }{\underset{n=1}{\sum }}A_{i,1}^{\omega_{i,n}}$$

and the standard deviation:

(6) $$\sigma_{\mu_{1}}=\sqrt{\frac{1}{\left(\alpha -1\right)\left(k-1\right)}\overset{\alpha }{\underset{n=1}{\sum }}\overset{k}{\underset{i=1}{\sum }}{\left(A_{i1}^{\omega_{in}}-{\bar{A}}_{\mu }\right)}^{2}}$$

In general, for any sub-locality $A_{i,j}^{\omega_{i,n}}$ associated with the star $S_{i}$, we can obtain the average of each star and the average of each set of random points of the module $A_{\mu_{j}}$. This average is:

$${\bar{A}}_{\mu_{j}}=\frac{1}{\alpha k}\overset{k}{\underset{i=1}{\sum }}\overset{\alpha }{\underset{n=1}{\sum }}A_{i,j}^{\omega_{i,n}}$$

and the standard deviation:

(7) $$\sigma_{\mu_{j}}=\sqrt{\frac{1}{\left(\alpha -1\right)\left(k-1\right)}\overset{\alpha }{\underset{i=1}{\sum }}\overset{k}{\underset{n=1}{\sum }}{\left(A_{i,j}^{\omega_{i,n}}-{\bar{A}}_{\mu_{j}}\right)}^{2}}$$

If we now fix a star, the average of areas and standard deviation of this locality by summation over α random set of points is

$${\bar{A}}_{\mu_{j}}\left(S_{i}\right)=\frac{1}{\alpha }\overset{\alpha }{\underset{n=1}{\sum }}A_{i,j}^{\omega_{i,n}}$$

and

(8) $$\sigma_{\mu_{j}}\left(S_{i}\right)=\sqrt{\frac{1}{\alpha -1}\overset{\alpha }{\underset{n=1}{\sum }}{\left(A_{i,j}^{\omega_{i,n}}-{\bar{A}}_{\mu_{j}}\left(S_{i}\right)\right)}^{2}}$$

The average of these standard deviations is calculated by performing summation over the k stars:

(9) $${\bar{\sigma }}_{\mu_{j}}=\frac{1}{k}\overset{k}{\underset{i=1}{\sum }}\sigma_{\mu_{j}}\left(S_{i}\right);\;\mathrm{Dispersion}\;\mathrm{mean}\;\mathrm{of}\;\mathrm{module}\;j;$$

which will have the final standard deviation:

(10) $$\sigma_{S\mu }=\sqrt{\frac{1}{\left(k-1\right)}\overset{k}{\underset{i=1}{\sum }}{\left(\sigma_{\mu_{j}}\left(S_{i}\right)-{\bar{\sigma }}_{\mu_{j}}\right)}^{2}};\;\mathrm{Standard}\;\mathrm{variation}\;\mathrm{of}\;\mathrm{dispersion}\;\mathrm{mean}\;\mathrm{of}\;\mathrm{module};$$

Modules associate the value ε with spatial organization since the variation of area sub-localties from two different values of eutacticity represents variation in module area for any sub-locality (Figure S1).

### 2.4. Standard Deviation of Partition Variability

In past Section 2.1, Section 2.2 and Section 2.3, we focused on a computational and quantitative method able to establish some important practical details concerning the measurement of planar spatial variations of shapes *Γ*. However, to unveil the geometrical properties that favor FO against any other symmetry, we can go beyond by proposing a numerical approach using partitions of planar discs (localities) divided into 3 to 10 sub-localities. In fact, this numerical experiment is necessary to relate Equations (1) and (2) with a proper collection of data reflecting a quantification of standard deviations of spatial organization in FO. A complete view of a wide spectrum of partitions of shapes *Γ* is obtained if we design a numerical model not restricted to the eutacticity parameter, since this parameter is proposed mainly as a tool but it is not a definite proof. Our geometrical design has as a first condition, the fact that planar discs with different numbers of sub-localities remains with a constant area during the experiment in order to have normalized data. Although we consider partitions of discs ranging from 3 to 10 sob-localities and each partition must be with a constant area during the experiment, we include 10 levels of variability. Therefore, each partition with particular constant area has 10 levels of variability during the experiment. According to Section 2.1, the standard deviation of each locality can be obtained by using Equation (2). For this purpose, we use Voronoi diagrams to model space partitioning with different number of parts (from 3 to 10), where two variables are studied, namely, partitioning number (pn) and partition variability (pv), which are defined as follows:

- Partitioning number (pn) defines the number of partitions inside a disc (ranging from three to ten).
- Partition variability (pv) determines multiple levels of variability (ten) inside discs by using random points, which in turn define the Voronoi diagrams. These levels of variability will be defined below.

**Figure 3:**
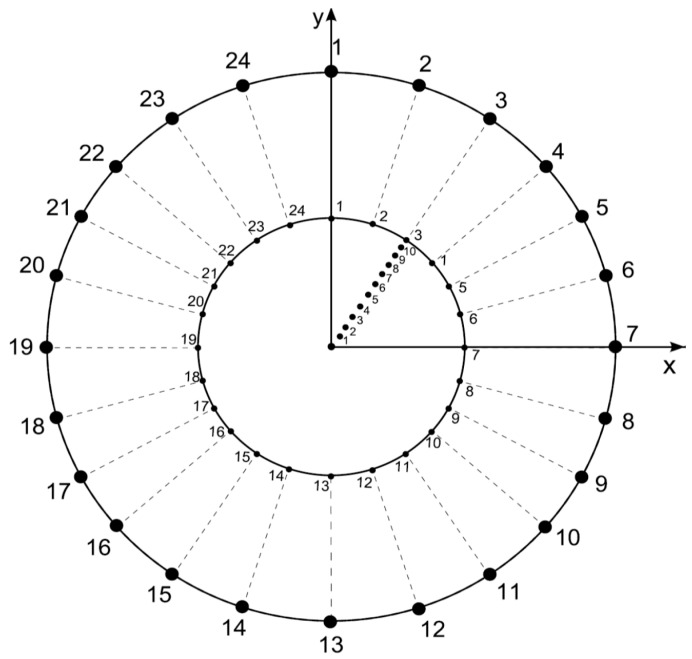
Defining partitioning number and partition variability. A disc is constructed to get Voronoi diagrams with constant area in spite of variability. The disc of this figure has a partitioning number of 2, one between axes x and y and the other is the remaining space. The magnitude of the radius defines ten levels of partition variability, which are the numbers emerging from the origin upon the diagonal on ray 3; (a) 1, (b) 1–2, (c) 1–3, (d) 1–4, (e) 1–5, (f) 1–6, (g) 1–7, (h) 1–8, (i) 1–9 and (j) 1–10. Each level of variability is given by radii ranges except (a) which is fixed at 1.

The algorithm to build partitioning and levels of variability of discs is described in the next seven steps as follows:

1. We initially consider a disk with a unitary radius where a second inscribed disk will be partitioned into a pn with a pv during the experiment (steps 4 and 5 of this algorithm, respectively). These discs are defined by particular features each: (a) The first disc is the external limit of the second and their coordinates are constant during the experiment; (b) the second one is constantly changing to obtain a pv (step 5 of this algorithm) and it is obtained by establishing a Voronoi tessellation. These two features (a) and (b) are described in the next steps (2) and (3) of this algorithm.
2. Features of external disc. The boundaries of the external limit are defined by 24 fixed points generated as follows: The radius of the external disk is set to *r* = 1 and consecutive points are separated by an angle *θ*/24. The functionality of this feature lies in the establishment of a fixed limit of reference to maintain a constant area during variation of partitions.
3. Features of internal disc. The boundaries of the internal limit are defined by 24 fixed points generated as follows: The radius of the internal disk is initially set to *r* = 0.53 ± 0.4 (established by the first level of variability step 6 of this algorithm) with 24 points consecutively separated by an angle *θ*/24. These radii are derived from a Voronoi tessellation whose points are the 24 points established before in this step besides the points derived from step 5. The functionality of this feature lies in the establishment of an internal limit able to change, providing statistical variation determining levels of variability of areas inside discs.
4. Now, we define partition numbering (pn) inside the disk. Once the number of partitions is defined, say n (where 3 ≤ n ≤ 10 and n∈Z) to define a Voronoi tessellation, points are located in the disk at angles 2π/n ± 0.069 radians but at different radius. These radius values will define the pv, as described in the next item.
5. Partition variability (pv). For each angular region defined above, 10 points are located at radius (between *r* = 0 and *r* = 10) at different positions to define different degrees of variability using Voronoi tessellations. The first point (first level of variability) is at *r* = 1. After the second point, all of them are located at random radius between 1 to 10. Hence, each level of variability (10) is given by radii ranges except 1 which is fixed at 1 (Figure 3); (a) 1, (b) 1–2, (c) 1–3, (d) 1–4, (e) 1–5, (f) 1–6, (g) 1–7, (h) 1–8, (i) 1–9 and (j) 1–10. Partition variability will define the broad spectrum of possibilities for area distribution inside discs without losing partitioning number. According to Equation (1), the average of areas requires a summation of sub-localities areas $\left(A_{ij}\right)$ which were derived from partitions.
6. Once the partition areas $\left(A_{ij}\right)$ inside discs were obtained and Equation (1) was solved, Equation (2) is used to get standard deviations $\left(\sigma_{i}\right)$ of variability for each disc. In order to normalize the level of variability for each pn, an index dividing the standard deviation of partitions and the particular area average of each partition was obtained (variability average; supporting information 2). There are eight particular area averages of partitions since we have a sample of 8 discs with different pn (from 3 to 10). These particular area averages are derived from a value n/(≈108.5 ± 1.5) which are n values obtained from the first level of variability (pv) at r = 1. It is important to say that the radius of the external disc (1) and the radius of the internal disc (*r =* 0.53 ± 0.4) was modified in order to get the particular area averages. However, in spite of the modification, the index between external discs and the internal ones remains constant. A sample of 20 discs to get 20 standard deviations $\left(20\;\sigma_{i}\right)$ was generated for each pn, and also for each level of pv (10) giving a sample of 200 discs for each pn. An average of standard deviations ($\bar{\sigma_{i}}$; variability average) was derived for each level of variability.
  Once the partition areas $\left(A_{ij}\right)$ inside discs were obtained and Equation (1) was solved, Equation (2) is used to get standard deviations $\left(\sigma_{i}\right)$ of variability for each disc. In order to normalize the level of variability for each pn, an index dividing the standard deviation of partitions and the particular area average of each partition was obtained (variability average; supporting information 2). There are eight particular area averages of partitions since we have a sample of 8 discs with different pn (from 3 to 10). These particular area averages are derived from a value n/(≈108.5 ± 1.5) which are n values obtained from the first level of variability (pv) at r = 1. It is important to say that the radius of the external disc (1) and the radius of the internal disc (*r =* 0.53 ± 0.4) was modified in order to get the particular area averages. However, in spite of the modification, the index between external discs and the internal ones remains constant. A sample of 20 discs to get 20 standard deviations $\left(20\;\sigma_{i}\right)$ was generated for each pn, and also for each level of pv (10) giving a sample of 200 discs for each pn. An average of standard deviations $(\bar{\sigma_{i}}$; variability average) was derived for each level of variability.
7. Finally, a standard deviation of all variability averages is obtained for each pn.

## 3. Results

### 3.1. Star Morphospace for Shapes Γ

**Figure 4:**
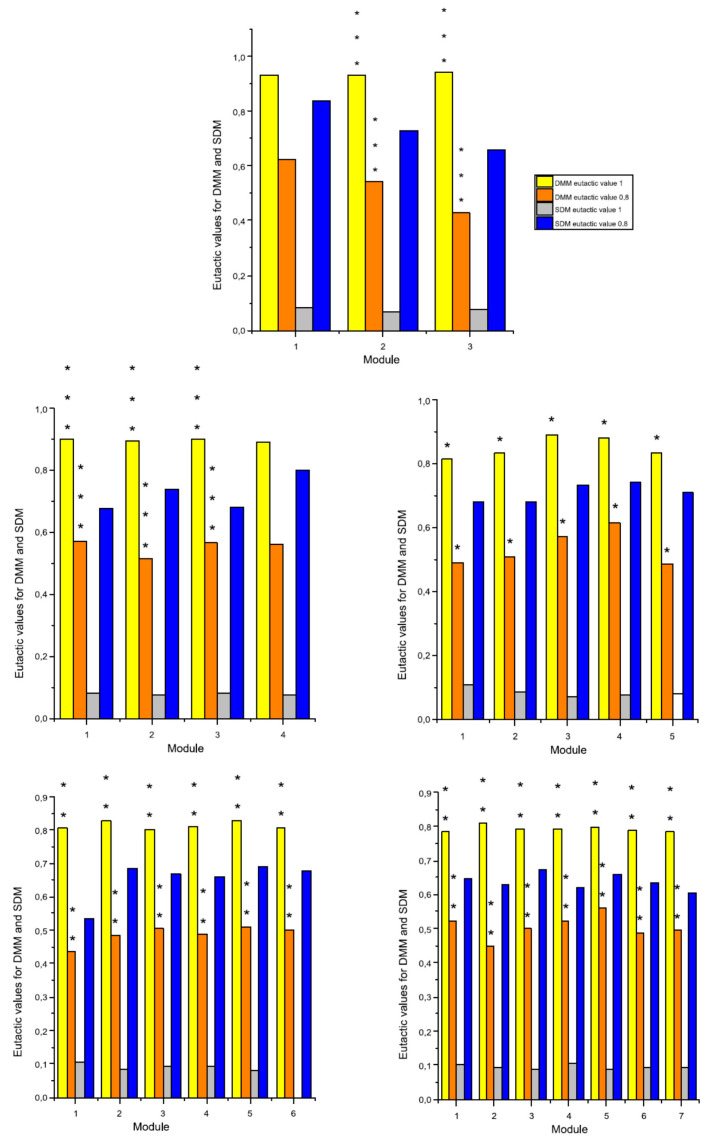
Dispersion mean of modules (DMM) and the standard variation of dispersion mean (SDM). DMM is the average of standard deviation of areas derived from Equation (9), from 100 localities using 100 sets of random points with several number of sub-localities with ε=1 ($\psi_{a}$; yellow bars) and ε=0.8 ($\psi_{b}$; orange bars). ANOVA test was performed in order to contrast eutactic values of DMM between $\psi_{a}$ and $\psi_{b}$. The obtained statistical significances of *p* range from less than 0.0001 for partitions with three modules and four modules (***); less than 0.05 for partitions with five modules (*); and less than 0.01 for partitions with six and seven modules (**). The null hypothesis was rejected in 23 of the 25 modules. The SDM (Equation (10)) for the module with ε=1 ($\psi_{a}$; grey bars) is notably smaller than the one obtained from the module with ε=0.8 ($\psi_{b}$; blue bars).

**Figure 5:**
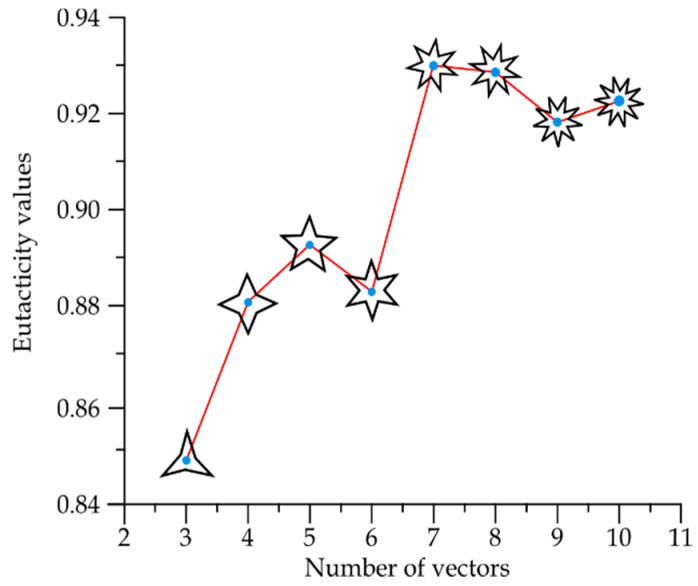
Star morphospace for eutacticity values derived from shapes *Γ*. Eutacticity means obtained from statistical distributions for vector stars ranging from 3 to 10 vectors.

Low values of eutacticty imply spatial heterogeneity while high values imply spatial homogeneity. Figure 4 shows that this standard deviation reflects the spatial variation of areas inside a given number of stars with $\varepsilon =1\left(\psi_{a}\right)$, in contrast with a second set of stars with $\varepsilon =0.8\left(\psi_{b}\right)$. Thus, the eutacticity parameter *ε* turns out to be useful to determine the spatial variation of areas inside localities (Equation (10)) when two values of eutacticity are compared. Using the idea of modules from Section 2.3 of methods we can conclude that the higher the eutacticity value, the higher the spatial homogeneity inside shapes, that is, the less the standard variation of dispersion mean (Equation (10); Figure 4). In other words, spatial heterogeneity increases according to the decreasing of eutacticity. In order to define particular values of this property, regarding spatial organization for statistical geometrical samples of several shapes *Γ* we must build that universe of shapes or star morphospace. Random stars (*n* = 10,000), with number of vectors *N* = 3, 4, 5, 6, 7, 8, 9 and 10 were generated according to a well established previous methodology reported in Reference [24]. Once these sets of stars are generated, eutacticity is measured in stars given eight particular statistical distributions (Figure S2). Those distributions are characterized by a mean which will give us a first insight about particular values of spatial organization for shapes *Γ* which will determine the resulting morphospace (Figure 5). As has already been mentioned in Section 2.2, in planar stars the range for eutacticity values is $\frac{1}{\sqrt{2}}\leq \varepsilon \leq 1$, which is a range between 0.7 and 1. A first interesting fact to highlight about distribution for eutactic values is that the mean value for stars with five vectors (0.89388) is higher than those values for both four and six vectors (0.88126 and 0.88324 respectively; Figure 5). As was expected from a first eye approach, over the statistical distribution for stars with three vectors (Figure S2a) the eutacticity value was lower (0.84827) than for all remaining stars (Figure 5). For stars above seven vectors, eutacticity values fall over 0.918. It is important to say at this point that stars with 8, 9 and 10 vectors can be considered as multiples of degree 2, 3, 4 and 5. However, it is not the case for stars with seven vectors. In fact, structures of seven folding order or higher are rare or absent, except those that can be considered as multiples of 2, 3, 4 and 5 [11]. Shapes with more than seven vectors can serve as controls to understand spatial deviations from the most abundant stars (3, 4, 5 and 6) and they will be included in our final analysis.

The resulting morphospace comes from mean eutacticity values derived from distributions of mean eutactic values (Figure S2). The establishment of a formal test comparing distributions was included in order to detect statistical differences between mean eutacticity values. We decided to contrast samples using a nonparametric statistical test, the Wilcoxon/Kruskall-Wallis test using the program JMP 8.0., since the statistical distributions are non-normal. The Wilcoxon/Kruskall Wallis standardized scores for three, four, five and six vector star distributions which fall below the mean while values for seven vectors or more are over the mean. Interestingly, scores for five vector stars is the nearest value to zero (Figure S3) which implies the most significative statistical difference. This fact reflects the increasing of the eutacticity mean for five vector stars visualized in the morphospace of Figure 5 in the middle of four and six vectors. In addition, four and six vector stars remain closest between them in contrast with five vector stars. Concerning this last point, we focused on comparing only four, five and six vector stars including a statistical analysis contrasting only these samples (Figure S4). This result shows how distribution of eutacticity values for five vector stars are considerably away from four and six vector star samples. According to this, we can conclude that eutacticity is a suitable measure able to detect variations of spatial organization inside polygons. The average for areas inside regular stars associated to highly eutactic stars reflects a tendency toward equal partition of internal space, while the high variation of SDM indicates that low eutactic stars have a much less equal distribution of areas. In that sense, statistically, five-folding stars are showing that they are in a particular position which is more regular than that for four-folding and six-folding organizations but less than organizations whose vectors are above seven vectors. 

### 3.2. Experimental Evidence

**Figure 6:**
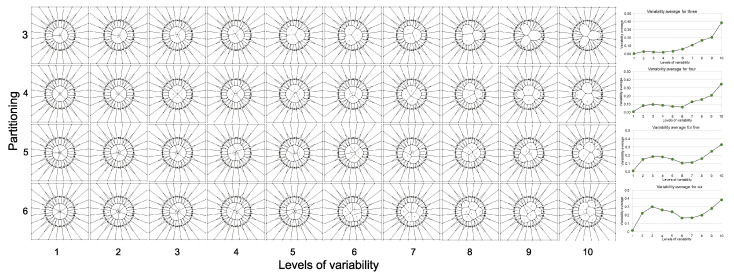
Partitioning number and partition variation of planar discs. A sample of 40 planar discs shows how partitioning number (vertical left side) determines segmentation of an almost constant area (≈108.5 ± 1.5) into a particular number of sub-localities. Partition variability (bottom horizontal numbers) installs levels of variability giving 10 constant and subtle increases of area to generate random segmentations. Variability averages (right vertical graphics) reflect the average of standard deviations ($\bar{\sigma_{i}}$) which is derived for each level of variability. It is important to note how each increase of variability enhances heterogeneity for every partitioning equally even if the graphics are dissimilar. Partitioning number for discs with 7, 8, 9 and 10 regions are showed in Figure S5.

**Figure 7:**
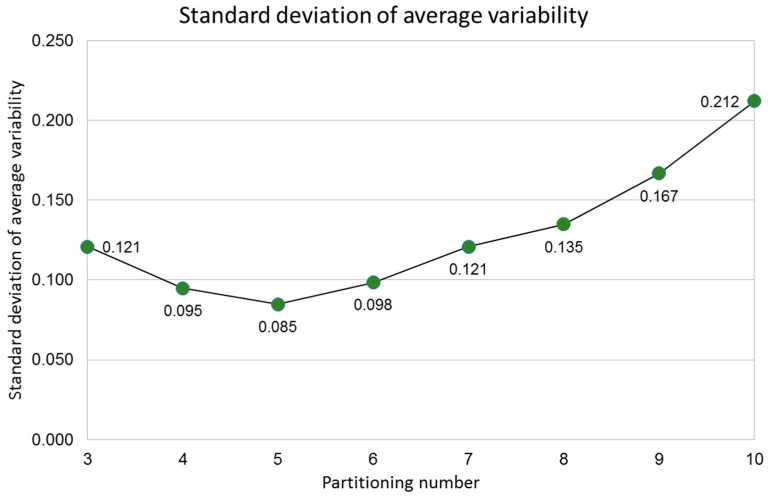
Standard deviation of all variability averages for each partitioning number. An average of standard deviations ($\bar{\sigma_{i}}$; variability average) was derived for each level of variability from Figure 6. A standard deviation of all variability averages is obtained for each partitioning number. According to this data, five-fold organizations are at the lowest level of dissimilarity among areas inside discs.

The final part of our methodology (Section 2.4) is based on a numerical approach determining particular values for partitions ranging from 3 to 10 sub-localities, using Equations (1) and (2). The experimental evidence in this section is devoid of eutacticity using a simulation of disc partitions regarding spatial variability. The results of this experiment are shown in Figure 6 and Figure 7. Each level of variability was composed of a sample of 20 standard deviations per partitioning (Figure 6). Spatial variability increases according to the levels of variability generating random points beyond the disc centroid which divides partitions enhancing Euclidean distance among random points. As an evidence of that spatial variability, we include eight graphics of averages of standard deviation plotting levels of variability (right side squares in Figure 6). Interestingly, the curves for variability averages between partitions are different. In addition, the lowest value for standard deviation of the overall sample of averages for standard deviation determined by the variability average is that for five partitioning number (Figure 7). Therefore, we can conclude that Equations (2) and (10) are appropriated to explain the selective frequency of FO in some natural systems since its selection is derived from a bias to equal spatial partitioning in spite of spatial variation.

## 4. Discussion

Low values of eutacticty imply spatial heterogeneity, while high values imply spatial homogeneity. According to our results, standard deviation of low eutactic stars are associated with an increase in spatial heterogeneity. Stars with eutactic values equal to *ε* = 1 (*ψ*_a_) remain in a zone with small SDM for modules, in contrast with those of a second set of stars with *ε* = 0.8 (*ψ*_b_). The morphospace of stars in Figure 5 shows that the mean eutactic value for stars with five vectors have a subtle major difference in contrast with stars with four and six vectors. Hence, two parameters are being important keys to detect spatial homogeneity inside morphospace, eutacticity and number of vectors. Whether dispersions of area distribution are associated with eutactic values we may conclude that small SDM for modules implies spatial homogeneity. Module variation implies fluctuating partitions without losing the particular correlation structure of the system related with polygonal side number. Therefore, the idea of modularity in our systems relies on a conserved polygonal structure even varying magnitudes of inner areas. One of our main hypotheses lies on considering these conserved polygonal structures as constraints that are not included as such, in our knowledge, in any other research. In that sense, polygons are changing in terms of spatial distribution depending on the number of vectors and the associated areas. Consequently, system-level properties for common architectures in simple polygonal forms are emergences of interacting spatial elements, attempting to gain space. One alternative, in terms of biological statistical mechanics, should be directed that endeavors as mechanical forces. However, according to our results, those forces might be considered as simple probabilistic parameters derived from standard deviations of modules immersed in polygons with a particular number of sides.

Section 3.2 of results includes experimental evidence of the outcome derived from star morphospace of Section 3.1 but is devoid of eutacticity. The idea of modules in this experimental evidence relies on two factors; partitioning number (number of partitions on each disc) and partition variability (levels of variability regarding levels of variation). According to this idea, there are system states such as irregular polygons or discs with particular numbers of regions which can distribute their inner space depending on the number of sides. That is, those several systems have fluctuating variations between areas which can be visualized with proper statistical tools. In fact, regular and symmetrical polygons whose inner space is not varying at all are not included as part of those system states since they are static entities neglecting variability. As a consequence, the experimental evidence exposed in Figure 6 and Figure 7 reflects that the lowest levels of spatial heterogeneity are for five-fold organizations. Despite the myriad of area variability inside discs of Figure 6, there are particular constraints in terms of standard deviations. The column on the right-side panel of Figure 6 shows different graphics for any partitioning number. In fact, Figure 7 exposes the global standard deviation of all variability averages for each partitioning number where five-fold organizations are at the lowest level of dissimilarity among areas inside discs. We assume as a possible hypothesis of these particular differences among the graphics for particular partitioning number differences among amounts of spatial heterogeneity. In order to achieve this novel context, we must visualize spatial homogeneity and spatial heterogeneity as probabilistic states whose levels of regularity can be related with particular system properties, such as biological behaviors or emergences. The defiance of some emergent structures to entropy would be behind a proper distribution of dynamical space. Some evolutionary processes, such as evolutionary convergences or selective events of some frequent architectures would be an outcome of modular internal arrangements of space, derived in the overwhelming amount of shapes, patterns and forms in nature, even in dynamic processes.

## 5. Conclusions

One of our global resulting conclusions is that the spatial organization for five-folding architectures or FO can be associated to a particular distribution of maximizing homogeneous internal space given by its geometry. That is, our idea lies on a suggestion that geometry defines a source of information and is not just a consequence of traditional physical button-up development. This last idea is notably different from those given by functional, ecological and even mechanical explanations because those hypotheses, traditionally, consider that form follows function. We consider that the significance of our findings is based on the statistical constancy of geometrical constraints, derived from the spatial organization of shapes beyond the material or complexity level of the many different systems. Our geometrical argument is not against the selective performance for five-folding symmetries in nature, since the high well qualified performance of this geometry during evolution could be a generic geometric constraint defined first as a system character before being a biological character.

## Figures and Tables

**Table 1:** Calculation of a module for sub-locality 1.

	Set of Random Points $\omega_{m,n}$ Defining the Associated Areas $A_{i,j}$ for Sub-Locality 1 (Algorithm Defined in Reference [24])					Summation of Areas for Star $\psi_{k}$
Stars	$\omega_{1,1}$	$\omega_{1,2}$	…	$\omega_{1,\alpha }$		
$\psi_{1}$	$A_{1,1}^{\omega_{1,1}}$	$A_{1,1}^{\omega_{1,2}}$	…	$A_{1,1}^{\omega_{1,\alpha }}$	⇒	$\frac{1}{\alpha }\overset{\alpha }{\underset{n=1}{\sum }}A_{1,1}^{\omega_{1,n}}$
$\psi_{2}$	$A_{2,1}^{\omega_{2,1}}$	$A_{2,1}^{\omega_{2,2}}$	…	$A_{2,1}^{\omega_{2,\alpha }}$	⇒	$\frac{1}{\alpha }\overset{\alpha }{\underset{n=1}{\sum }}A_{2,1}^{\omega_{2,n}}$
. . .	. . .	. . .	. . .	. . .	. . .	. . .
$\psi_{k}$	$A_{k,1}^{\omega_{k,1}}$	$A_{k,1}^{\omega_{k,2}}$	…	$A_{k,1}^{\omega_{k,\alpha }}$	⇒	$\frac{1}{\alpha }\overset{\alpha }{\underset{n=1}{\sum }}A_{k,1}^{\omega_{k,n}}$

## Supplementary Materials

Supplementary materials are available at http://www.mdpi.com/1099-4300/20/9/705/s1.
