# Supplementary material for: Spatial Organization of Five-Fold Morphology as a Source of Geometrical Constraint in Biology
Source: Entropy (Basel). 2018 Sep 14;20(9):705. doi: 10.3390/e20090705 (PMC7513224; doi:10.3390/e20090705)
Supplement: Supplementary file 1 [file entropy-20-00705-s001.pdf]

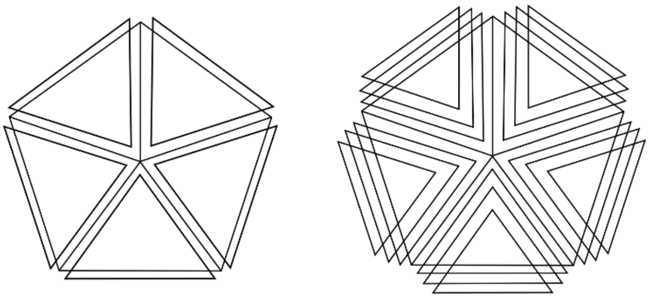

(**a**) (**b**)

Figure S1. Scheme for area variability of modules defined by regular stars (**a**) versus modules defined by irregular stars (**b**) ones. In case (**a**), low variability in areas (spatial homogeneity derived from standard variation of dispersion mean of module; equation 10) is depicted; this low variability implies non-significant overlap between modules. On the contrary, in case (**b**) high variability in areas (spatial variability derived from standard variation of dispersion mean of module) is depicted; it implies significant overlap between modules or high variation.

Table S1

**Partitioning numbering**	**Area at internal disc (level of variability pv1)**	**Particular area average**
**3**	**107.2**	35.7354
**4**	108.7	27.1963
**5**	109.5	21.9155
**6**	109.9	18.3248
**7**	110.1	15.74
**8**	110.32	13.7959
**9**	110.51	12.2794
**10**	110.605	11.0605

Table: In order to normalize the level of variability for each pn, an index dividing the standard deviation of partitions and the particular area average of each partition was obtained (variability average)**.** There are eight particular area averages of partitions since we have a sample of 8 discs with different pn (from 3 to 10). These particular area averages are derived from a value n/(≈108.5±1.5) which are n values obtained from the first level of variability (pv) at r=1. It is important to say that the radius of the external disc (1) and the radius of the internal disc (*r=0.53*±0.4) was modified in order to get the particular area averages. However, in spite of the modification the index between external discs and the internal ones remains constant. A sample of 20 discs to get 20 standard deviations (20 σ_i) was generated for each pn, and also for each level of pv (10) giving a sample of 200 discs for each pn. An average of standard deviations ($\overline{\sigma_i}$; variability average) was derived for each level of variability.

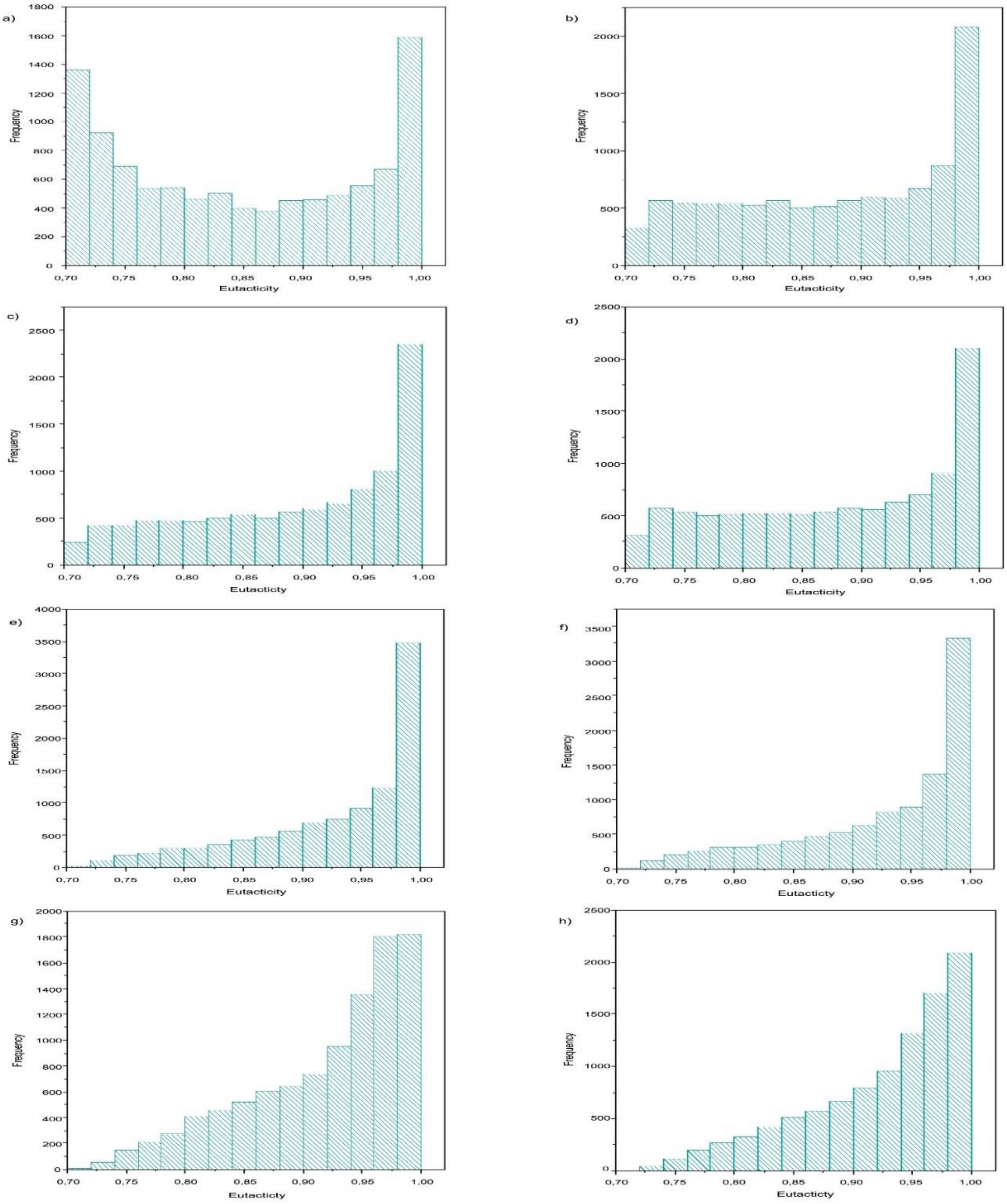

Figure S2. Statistical distributions for random stars showing frequency of eutacticity values. The abscissa indicates eutacticity values (ranging from 0.7 to 1), and ordinate is frequency for vector stars ranging from 3 to 10 vectors (letters from a=3, b=4, c=5, d=6, e=7, f=8, g=9 and h=10).

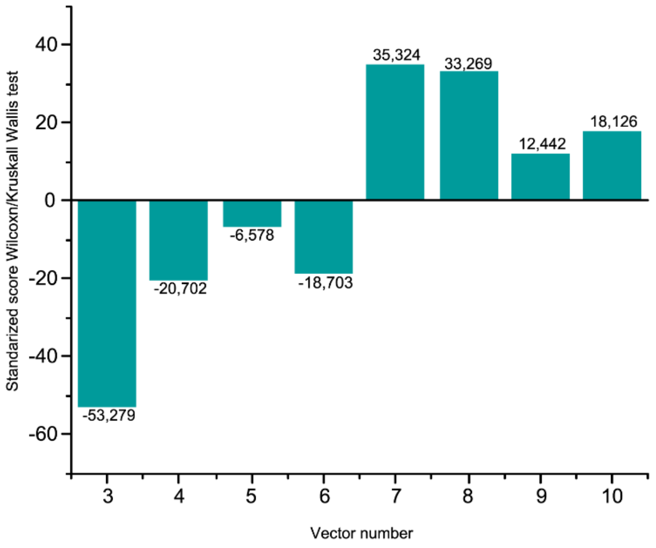

Figure S3. Standardized score Wilcoxon/Kruskall Wallis for vector stars ranging from 3 to 10 vectors. Score for five vector stars is the closest value to zero.

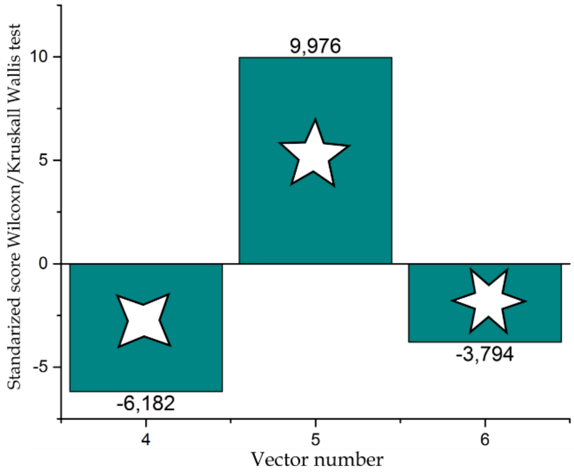

Figure S4. Standardized score Wilcoxon/Kruskall Wallis for vector stars ranging from 4 to 6 vectors. According to the standardized score Wilcoxon/Kruskall Wallis test five vector stars are statistically dissimilar to four and six vectors distributions.

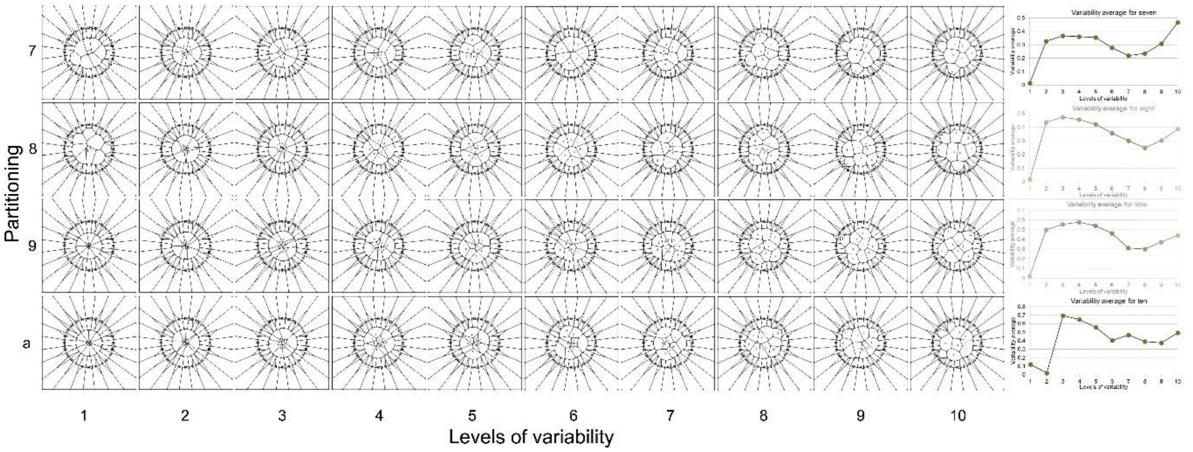

Figure S5. Partitioning number and partition variation of planar discs. A sample of 40 planar discs shows how partitioning number (vertical left side) determines segmentation of an almost constant area (≈108.5±1.5) into particular number of sub-localities. Partition variability (bottom horizontal numbers) installs levels of variability giving ten constant and subtle increases of area to generate random segmentations. Variability averages (right vertical graphics) reflects average of standard deviations ((σ_i) ̅) which is derived for each level of variability. It is important to note how each increase of variability enhance heterogeneity for every partitioning equally even the graphics are dissimilar.
